# Reply to Kloepfer and Gern: Independent studies suggest an arms race between influenza and rhinovirus: What next?

**DOI:** 10.1073/pnas.2000903117

**Published:** 2020-03-03

**Authors:** S. Nickbakhsh, C. Mair, L. Matthews, R. Reeve, P. C. D. Johnson, F. Thorburn, B. von Wissmann, J. McMenamin, R. N. Gunson, P. R. Murcia

**Affiliations:** ^a^Medical Research Council–University of Glasgow Centre for Virus Research, Institute of Infection, Immunity and Inflammation, College of Medical, Veterinary and Life Sciences, University of Glasgow, Glasgow G61 1QH, United Kingdom;; ^b^School of Mathematics and Statistics, College of Science and Engineering, University of Glasgow, G12 8QQ Glasgow, United Kingdom;; ^c^Boyd Orr Centre for Population and Ecosystem Health, Institute of Biodiversity, Animal Health and Comparative Medicine, College of Medical, Veterinary and Life Sciences, University of Glasgow, Glasgow G12 8QQ, United Kingdom;; ^d^The Queen Elizabeth University Hospital, National Health Service Greater Glasgow and Clyde, Glasgow G51 4TF, United Kingdom;; ^e^Public Health, National Health Service Greater Glasgow and Clyde, Glasgow G12 0XH, United Kingdom;; ^f^Health Protection Scotland, National Health Service National Services Scotland, Glasgow G2 6QE, United Kingdom;; ^g^West of Scotland Specialist Virology Centre, National Health Service Greater Glasgow and Clyde, Glasgow G31 2ER, United Kingdom

It was very interesting to learn about Kloepfer et al.’s study investigating the link between asthma and influenza A H1N1 infection incidence and severity (1). Their finding that influenza A virus (IAV) infections reduced the subsequent risk of infection with human rhinoviruses (HRVs) was contrary to theories at the time of a reverse directionality (2, 3). However, as Kloepfer and Gern (4) state in their Letter, it is certainly consistent with our recent large-scale study on respiratory virus–virus interactions (5). Our study primarily aimed to provide statistical evidence for interactions between 11 groups of influenza and noninfluenza viruses using a bespoke statistical methodology for investigation of time series correlation at the population scale (6). Uniquely, we were able to concurrently evaluate inferences at the individual host scale, made possible by the simultaneous testing of patients for multiple respiratory viruses.

However, as Kloepfer and Gern (4) highlight, an important limitation of our study is that routine diagnostic data reflect a single snapshot of an individual’s infection. Our host-scale analysis, quantifying relative risks of virus codetections in the presence/absence of other viruses, provided strong support for a negative association between IAV and HRV (odds ratio = 0.27, 95% CI = 0.14 to 0.51, *P* < 0.001). However, the sequential timing of infection events, and therefore the directionality of effect, could not be directly determined from our data.

The prospective longitudinal study employed by Kloepfer et al. (1), on the other hand, although based on a comparatively small study of children sampled over a short 2-mo period, does provide suitable data to infer directionality. Their study supports a unidirectional relationship, as we had hypothesized based on epidemiological reasoning. We examined this hypothesis in mathematical simulations and found that interference with IAV could cause a measurable decline in HRV incidence in winter, as we observed empirically (5).

Moving forward, several important knowledge gaps remain surrounding mechanisms and generality of IAV–HRV interaction. First, is the form of interaction (negative), magnitude, and directionality of effect consistent across H1N1 and H3N2 influenza subtypes and HRV species and serotypes? Second, is the nature of virus–virus interactions altered by comorbidities such as asthma, chronic obstructive pulmonary disease, or other immune disorders? Last, how localized or spatially widespread is the existence and the nature of IAV–HRV interaction? Ultimately, a collaborative effort spanning multiple scientific disciplines is needed to establish the cellular-level mechanism(s) of interference, the impact on the within-host dynamics of infection, and the evolutionary drivers underpinning this battle for coexistence in the human respiratory tract.
